# Controversial significance of early S100B levels after cardiac surgery

**DOI:** 10.1186/1471-2377-4-24

**Published:** 2004-12-16

**Authors:** Henrik Jönsson, Per Johnsson, Martin Bäckström, Christer Alling, Cecilia Dautovic-Bergh, Sten Blomquist

**Affiliations:** 1 Department of Heart-Lung Diseases, Lund University Hospital, Lund, Sweden; 2 Departments of Psychology, Lund University, Lund, Sweden; 3 Department of Medical Neurochemistry, Lund University Hospital, Lund, Sweden

## Abstract

**Background:**

The brain-derived protein S100B has been shown to be a useful marker of brain injury of different etiologies. Cognitive dysfunction after cardiac surgery using cardiopulmonary bypass has been reported to occur in up to 70% of patients. In this study we tried to evaluate S100B as a marker for cognitive dysfunction after coronary bypass surgery with cardiopulmonary bypass in a model where the inflow of S100B from shed mediastinal blood was corrected for.

**Methods:**

56 patients scheduled for coronary artery bypass grafting underwent prospective neuropsychological testing. The test scores were standardized and an impairment index was constructed. S100B was sampled at the end of surgery, hourly for the first 6 hours, and then 8, 10, 15, 24 and 48 hours after surgery. None of the patients received autotransfusion.

**Results:**

In simple linear analysis, no significant relation was found between S100B levels and neuropsychological outcome. In a backwards stepwise regression analysis the three variables, S100B levels at the end of cardiopulmonary bypass, S100B levels 1 hour later and the age of the patients were found to explain part of the neuropsychological deterioration (r = 0.49, p < 0.005).

**Conclusions:**

In this study we found that S100B levels 1 hour after surgery seem to be the most informative. Our attempt to control the increased levels of S100B caused by contamination from the surgical field did not yield different results. We conclude that the clinical value of S100B as a predictive measurement of postoperative cognitive dysfunction after cardiac surgery is limited.

## Background

Despite the fact that incidence figures between 4–79% have been reported for cognitive dysfunction after cardiac surgery [1], diagnostic steps are seldom taken to diagnose this impairment. The golden standard for detecting cognitive dysfunction is neuropsychological tests, which are complex and difficult to use as a routine procedure. Lately the brain derived protein S100B has been proposed as a simple method for detecting brain dysfunction after cardiac surgery [2-5]. The protein is a member of the larger S100 family, where S100B is one isoform, and considered to be brain specific, and the other is the S100A isoform [6]. The S100B chain is uniform in contrast to the S100A chain which exhibits several subgroups. The dimers of interest in studies concerning cerebral events are those containing S100B (S100BB and S100A1B). The physiological roles of S100B are pleiotropic including neurotrophic and neuroprotective functions, mediated by calcium dependent regulation of phosporylation, enzyme activation and proliferation [6]. On the other hand, high concentrations of S100B have been shown to be toxic and induce apoptosis in neuronal cell cultures [7,8]. At least five experimental studies have indicated a possible role of S100B in learning and memory function, three of which reported impaired memory and learning effects in transgenic S100B mice and two reported memory deficits after injection of S100B antiserum [9-13].

Several studies in humans suffering from stroke of different ethiologies, have shown a rather strong correlation between serum levels of S100B and size of lesion(s) as well as outcome [14-17].

Lately a number of studies have addressed the question whether S100B can be considered as a marker for cognitive dysfunction after cardiac surgery; however the conclusions presented are disparate. One major reason for this could be the fact that S100B is present in high concentrations in shed mediastinal blood that is retransfused to the patient by cardiotomy suction and autotransfusion, thus obscuring the measured levels of S100B early after surgery. To date, only one study have been published where this contamination was taken into account [18]. We recently reported the half life of S100B in serum to be 25 min [19]. With the present study we wanted to use our knowledge of inflow and elimination to obtain a more reliable measurement of cerebral release of S100B after cardiac surgery and correlate it with neuropsychological outcome.

## Methods

### Study design

The study group comprised 56 patients who underwent coronary artery bypass graft (CABG) surgery at the Division of Cardiac Surgery, University Hospital MAS, Malmoe, Sweden and Department of Cardiothoracic Surgery, Lund University Hospital, Lund, Sweden. Only patients planned for elective CABG with cardiopulmonary bypass (CPB) as their sole procedure were included. The study protocol was approved by the local ethics committee, and patients gave a written informed consent before the study protocol was initiated. Patients with a history of stroke, transient ischemic attack (TIA), reversible neurological disorder (RIND), known carotid artery disease or other brain diseases were excluded. In order to avoid possible influence of renal disorder on the elimination of S100B, patients with known renal failure (Creatinin > 160 μmol/L) were excluded.

The patients were examined for signs of neurological dysfunctions daily during the hospital stay by either experienced cardiac anesthesiologists or by experienced cardiac surgeons.

### Perioperative management

Anesthesia was induced with midazolam 3–5 mg iv (Dormicum^®^, Roche, Basel, Switzerland) or propofol (Diprivan^®^, Zeneca Ltd, Cheshire, England) 10 mg/kg. It was subsequently maintained with fentanyl 10 μg/kg (Leptanal^®^, Janssen Pharmaceutica, Beerse, Belgien), a continuous infusion of propofol 3–6 mg/kg/h or inhalation of isoflurane 0,5–1% (Forene^®^, Abbott Laboratories). Nitrous oxide (Aga Industries, Stockholm, Sweden) was used before CPB but not during or after CPB.

CABG surgery was performed during aortic cross clamping with the distal anastomosis preceding the proximal anastomosis. A tangential occluder replaced the cross-clamp during the proximal anastomosis. Antegrade cold S:t Thomas crystalloid cardioplegia was used (Cardioplegi^®^, Pharmacia-Upjohn, Uppsala, Sweden) and administered in the ascending aorta and the anastomosed vein-grafts intermittently.

Perfusion was performed with a roller pump (Cobe Industries, Denver, Colorado, USA). The perfusion catheters and circuit were made of polyvinylchloride in the line and silicon in the pumphead. The arterial cannulation was made in the ascending aorta and venous cannulation in the right atrium by a two-stage venous cannula. All circuits contained a heparin-coated 40 μm arterial filter (Cobe Sence, Cobe Industries) and a membrane oxygenator (Cobe Duo oxygenator, Cobe Industries). The circuit was primed with approximately 1000 ml of Ringer's lactate (Pharmacia-Upjohn), 250 ml 15% Mannitol (Pharmacia-Upjohn) and 75 mmol Addex tromethamine (Pharmacia-Upjohn). Perfusion flow was non-pulsatile with a flow rate of 2.4 l/min/m^2 ^at normothermia. The perfusate was cooled to approximately 32°C. Heparin (400 U/kg bodyweight) was given prior to cannulation and reversed with equal doses of protamine sulphate at decannulation.

After surgery, the patients patient were transferred to the ICU for recovery and enrolled in the sampling scheme for S100B analysis. None of the patients received autotransfusion.

### S100-protein analysis and calculations

Serum for S100B analysis was sampled before surgery, at the end of CPB, and then 1, 2, 3, 4, 5, 6, 8, 10, 15, 24 and 48 hours after surgery. The S100B levels at these time points will be referred to as T0, T1, T2....T48. Blood samples, both arterial and venous samples, were cooled and centrifuged within 5 hours. All samples were measured by a monoclonal two-site immunoluminometric assay (Sangtec 100, AB Sangtec Medical, Bromma, Sweden).

### S100B kinetic calculations

**Figure 1:**
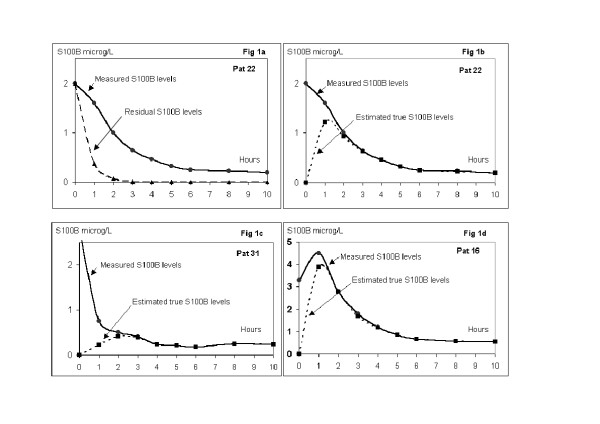
1a – Measured S100B release pattern in one patient and the calculated residual levels from the S100B from cardiotomy suction during surgery, a half-life of 25 minutes was used. 1b – Estimated true release, calculated by subtracting the residual levels (from 1a) from measured levels. 1c – Measured levels and estimated true release from one patient with high S100B at T0 and low estimated true release at T1. This patient also had a good neuropsychological outcome. 1d – Measured levels and estimated true release from one patient with low S100B at T0 and high estimated true release at T1. This patient had a bad neuropsychological outcome.



Since early levels of S100B are contaminated by S100B from cardiotomy suction, an attempt was made to exclude this S100B from the levels measured one and two hours after surgery. Assuming that all of the measured S100B at the termination of CPB was a contamination, this non-cerebral S100B was eliminated with a half-life of 25 minutes, as illustrated in figure 1a and 1b. The estimated true levels cleansed from the contamination 1 and 2 hours after the end of surgery were thereby calculated by the formula:

where C_e _is the estimated true levels of S100B at time t, C_t _= the measured concentration of S100B at T1 or T2, C_0 _the concentration at the end of CPB, t the time after T0 and t_1/2 _the half-life of S100B.

We have earlier suggested that the elimination rate could be used as another measure of cerebral release [5], and the elimination rates of S100B between the end of CPB (T0) and 1 hour later, between T1 and T2, and between T2 and T3 were calculated accordingly. The differences between measured S100B levels at T0 and T1, T0 and T2 were also calculated.

### Neuropsychological method

The patients underwent neuropsychological testing by the same trained neuropsychologist 1–2 days before and 5–7 days after surgery. The tests used were: Mental Control, Figural Memory, Logical Memory (A/B), Visual Reproduction, Rey Auditory/Verbal Learning Test (RAVLT), Trail Making A, Trail Making B, Digit Symbol, Digit Span, Visual Memory Span, Visual Paired Associates II or Verbal Paired Associates I and RAVLT, Delayed Retention. The tests were chosen from the Wechsler Memory Scale-Revised (WMS-R), the Wechsler Adult Intelligence Scale (WAIS – R) and the Halsted-Reitan Neuropsychological Battery [20,21].

Differences for each sub-test were first calculated and then standardized to z-values. All sub-tests were then aggregated to create an impairment index [4]. The impairment index was a continuous variable, where a positive value reflected an improvement in neuropsychological test and a negative value reflected deterioration. The incidence of neuropsychological impairment in the study group was calculated according to the 1 standard deviation criterion (SD) as defined by Newman and the 20% criterion as defined by Stump [22-24].

### Statistical analysis

All results were analyzed with the Statistica version 5.0 for PC. Regression analysis was performed with the least square method with a casewise deletion of missing data. For the multiple regression a backwards stepwise regression was performed to determine which variables to include in the final analysis. The initial variables included in this analysis were age, gender, perfusion time, years of education, and S100B levels at all sampling times. A p-value < 0,05 was considered significant. Unless otherwise stated numerical values are presented as mean ± 1 standard deviation.

## Results

Patient demographics are presented in table 1. None of the patients suffered a clinically detectable stroke. The incidence of neuropsychological impairment was 37,5% (n = 21, 95% C.I 26,0–50,6%) according to the 1 standard deviation criterion, and 80,4% (n = 45, 95% C.I. 68,1–88,6%) according to the 20% criterion.

**Table 1:** Demographics for the study group.

	Mean	St. dev.
Number	56	
Sex (M/F)	47/9	
Age (years)	60.4	9.0
Education (years)	9.6	3.1
Perfusion time (Minutes)	82.8	31.5
X-clamp (minutes)	53.9	23.5

**Figure 2:**
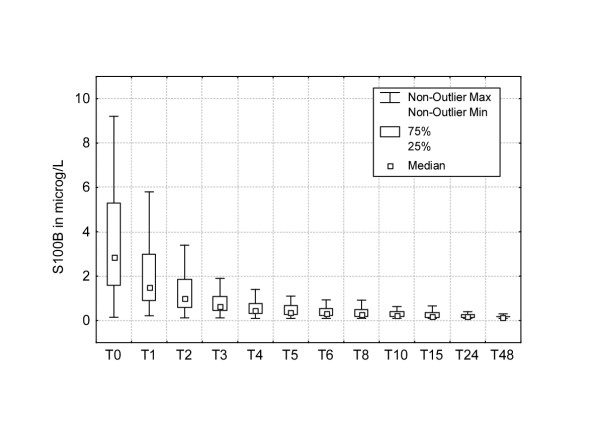
S100B release pattern after cardiac surgery with cardiopulmonary bypass shown as a boxplot.

The appearance of S100B protein followed the same pattern in all patients with high levels at the end of CPB and a decrease or slight increase the first hour (figure 2). Thereafter a subsequent decrease was observed during the rest of the study period, except in one patient in whom the concentration of S100B increased to 2,0 μg/L 48 hours after surgery. Mean estimated true release of S100B, calculated according to equation [1], was lower at T1 and T2 compared to measured S100B levels (1.43 ± 1.37 μg/L vs. 2.11 ± 1.81 μg/L and 1.22 ± 1.10 μg/L vs. 1.32 ± 1.17 μg/L). Three representative examples of this calculation are shown in figure 1b, 1c and 1d.

We found a correlation between patient age and S100B levels measured up to 24 hours after bypass (Table 2). No significant correlation was found between measured S100B levels at the sampling times and neuropsychological impairment in simple regression analysis (Table 3). Neither did age, years of education and duration of perfusion correlate with neuropsychological impairment when tested in simple regression analysis (Table 3).

**Table 2:** The relation between S100B levels and patient age tested in univariate linear regression analysis.

	r-value	p-level
S100B at T0	0.19	n. s
S100B at T1	0.30	<0.05
S100B at T2	0.30	<0.05
S100B at T3	0.34	<0.05
S100B at T4	0.37	<0.05
S100B at T5	0.32	<0.05
S100B at T6	0.37	<0.05
S100B at T8	0.38	<0.01
S100B at T10	0.42	<0.005
S100B at T15	0.36	<0.05
S100B at T24	0.46	<0.005
S100B at T48	0.004	n.s

**Table 3:** The relation between S100B levels, demographic variables and neuropsychological impairment index, expressed as r-value from linear regression analysis (* = p < 0.05). Mean (± SD) S100B levels are also presented. (Est. true release = estimated true release when residual levels from contamination have been excluded)

	Mean ± St.dev.	r-value
S100B at T0	3.63 ± 2.84	0.08
S100B at T1	2.11 ± 1.81	-0.16
S100B at T2	1.32 ± 1.17	-0.11
S100B at T3	0.87 ± 0.73	-0.12
S100B at T4	0.60 ± 0.42	-0.06
S100B at T5	0.49 ± 0.38	-0.12
S100B at T6	0.42 ± 0.33	-0.15
S100B at T8	0.38 ± 0.25	-0.12
S100B at T10	0.31 ± 0.21	-0.06
S100B at T15	0.25 ± 0.16	-0.09
S100B at T24	0.21 ± 0.11	-0.10
S100B at T48	0.21 ± 0.27	-0.09

Est. true release at T1	1.43 ± 1.37	-0.22
Est. true residual at T2	1.22 ± 1.10	-0.12

Elimination rate T0-T1	0.50 ± 0.46	0.26
Elimination rate T1-T2	0.48 ± 0.25	-0.05
Elimination rate T2-T3	0.68 ± 0.32	-0.12
Difference T0-T1	1.53 ± 1.66	0,30*

Difference T0-T2	2.31 ± 2.01	0,18

Age		0.16
Education		-0.06
Perfusion time		0.12

In multiple regression analysis, measured S100B levels at the end of CPB (T0), one hour later (T1) and age were found to explain part of the neuropsychological impairment (r = 0.49, p < 0.005, Table 4). It is worth noting that the correlation was positive at T0 and negative at T1, implicating a better outcome if S100B was high at T0 and worse if the levels were high at T1. Age had a negative correlation. Furthermore the combination of age, S100B at T0 and S100B at either T3 or T6 gave significant correlations in the same manner (r = 0.41, p < 0.05 and r = 0.42, p < 0.05 respectively). The difference between S100B levels at T0 and T1 was found to correlate positively with neuropsychological outcome (Table 3). No correlation was found when the estimated true release of S100B was tested against neuropsychological outcome, neither did the elimination rate fall out significantly in simple regression analysis (Table 3).

**Table 4:** The results of the backwards stepwise multiple regression analysis model to explain the neuropsychological impairment index (r = 0.49, p < 0.005).

Variable	Partial correlation	Beta	p-value
S100 at T0	0.45	0.845	<0.005
S100 at T1	-0.47	-0.939	<0.001
Age	0.31	0.307	<0.05

Regression			<0.005

## Discussion

This study presents some interesting findings that may be relevant in clinical practice. There is a clear relation between patient age and release of S100B up to 24 hours after cardiac surgery. This correlation is strong and not influenced by the perioperative contamination of S100B caused by the use of coronary suction [4].

The univariate analysis did not provide a clear answer to the question whether there is a relationship between neuropsychological outcome and S100B release. However, from table 3 it is worth noting that the r-values in univariate regression analysis are negative at all sampling points except at T0 (immediately after bypass). The consistent finding of a negative correlation 1 hour and onwards after bypass is intriguing and warrants further exploration.

The multiple regression analysis resulted in a significant correlation between neuropsychological deterioration and the three variables: S100B at T0, S100B at T1 and patient age. The r-value in the multiple regression analysis was 0.49, which evokes the question why the multiple regression is stronger than the univariate regression. One possible explanation could be that by including the S100B level at the end of CPB we compensate for some part of the contamination in the S100B levels one hour after surgery.

Age correlated also with outcome. As expected, the older the patient the higher the risk for neuropsychological deterioration, which is in accordance to the fact that age is a risk factor for decline in neuropsychological tests after surgery [23].

Our results are to some extent in concordance with other reports in this field. Two groups have reported no relation between S100B and neuropsychological test results, [2,18] and two groups have found a relation using a composite S100B end-point [3,4]. Only one of these studies was designed to exclude the extracerebral inflow of S100B [18]. These contradicting findings support our notion that S100B kinetics after cardiac surgery are complicated.

Interestingly, in the multiple regression analysis there was a positive correlation between S100B levels at T0 and outcome, a finding that stands in contrast to the hypothesis that S100B could be used as serum marker for cognitive dysfunction. This finding is especially interesting since most of the measure S100B at T0 is contamination. In an earlier study we found that approximately 80% of measured S100B at this point is from extracerebral sources in CABG patients [4]. We offer no explanation for this unexpected finding, but it is intriguing and calls for further investigations.

When interpreting the results of our study, as well as comparing them with those of other reports, several issues need to be addressed. To begin with the origin of S100B measured after CPB is not exclusively cerebral. From our previous study as well as from reports by Anderson et al [25], it is clear that shed blood in the mediastinum contains very high levels of S100B. When this blood is retransfused either directly by cardiotomy suction or later in the course by autotransfusion during the postoperative care, the systemic levels of S100B are affected. The source of this S100B is non-cerebral, probably from S100B containing tissue such as fat, skin and bone marrow. No autotransfusion was used in this study, however reabsorbtion of S100B from injured fat tissue may contribute to the measured serum levels of S100B.

Since we previously have been able to determine the half life of S100B in blood to be 25 min, it was possible to control for the peri-operative contribution from cardiotomy suctions by using the equation mentioned to obtain the estimated true release at T1 and T2. When these values were entered in simple regression analysis, no correlation was found to neuropsychological outcome. However, if multiple regression analysis was used, a positive correlation was found, these results were identical to those obtained with the measured S100B values, and therefore we can conclude that the use of the correction procedure represented by the equation does not contribute to the evaluation of the results. Moreover, we should be careful to overinterpret the significance of correlations in a study of relatively few patients and several end-points, i.e sampling times for S100B. There is always a risk of accepting a false hypothesis. Preferably, a study of this sort should contain a large number of patients and only one or few end-points. However, the aim of this study was to clarify the complex kinetics of early S100B release and its possible connection to a neuropsychological decline, not to determine the perfect use of S100b for detecting neuropsychological outcome after cardiac surgery.

In the mathematical correction model suggested here, we assume that all S100B measured at T0 is of extrecerebral origin. This is of course not the case, but we do not know the relation between cerebral and extracerebral S100B at this time-point in each individual. By assuming that none of the S100B measured at T0 is of cerebral origin, we can be sure that our mathematical model does not include non-cerebral S100B at T1 and later. However, these levels might be lower than the true levels of cerebral S100B since we could have excluded cerebral S100B present at T0. This could also be an explanation that the multiple regression analysis showed stronger correlations.

One important issue, which must be considered in this type of study, is the strength that can be expected in the correlation between two different measures of brain function. The functional domains in the brain covered by neuropsychological tests vary depending on the tests used and are by no means complete in any test battery. By the same reasoning, the magnitude of a possible cerebral release of S100B after an injury may vary according to the severity of the insult as well as the location of the insult, since glia dense areas express more S100B than others [26].

## Conclusions

In conclusion, with the present knowledge, a single S100B sampled in the postoperative course after cardiac surgery can not be of use in clinical practice in order to predict neuropsychological outcome with an acceptable sensitivity.

However, utilizing a statistic model, an association between S100B levels the first hours after surgery and neuropsychological outcome was found, where the most informative time point seems to be 1 hour after the termination of CPB.

The controversial significance of an increased S100B immediately after surgery is indeed intriguing and it inspires further studies of the mechanisms of S100B release after cardiac surgery as well as other fields of brain damage.

## Competing interests

The author(s) declare that they have no competing interests.

## Authors' contributions

HJ: Principal investigator recruited, enrolled patients analyzed and wrote the paper

PJ: Had an integral role in the planning and the analysis, also help with writing

MB: Designed neuropsychological test battery. Analyzed neuropsychological data

CB: Performed all neuropsychological testing with patients

CA: Did the S100B-analyzes

SB: Mentor an principal leader of the project, who also helped with the writing.

## Pre-publication history

The pre-publication history for this paper can be accessed here:


